# Maria Asuncion Silvestre: improving neonatal care

**DOI:** 10.2471/BLT.23.030823

**Published:** 2023-08-01

## Abstract

In the run-up to World Breastfeeding Week, Maria Asuncion Silvestre talks to Gary Humphreys about supporting and protecting exclusive breastfeeding as part of evidence-based neonatal care.

*Q: How did you come to focus on the breastfeeding issue?*

A: After graduating from medical school, I specialized in paediatrics, so naturally took a keen interest, but having grown up in a household where breastfeeding was part of the daily routine, I think I went into my studies with a certain mindset. My mother, who was a nurse, exclusively breastfed me and my six siblings and, although I didn’t pay much attention at the time, it was part of my reality growing up. I did my paediatric residency in the Philippines and completed advanced training in neonatal and perinatal medicine at hospitals in the USA. When I returned to the Philippines in 1987, I went to work at the University of the Philippines College of Medicine, where I served as an attending neonatologist and, eventually, an associate professor. I thus had a front row seat at hundreds of births and interacted with mothers, many of whom were either very young or had come through challenging pregnancies and deliveries. I learned a lot about the importance of establishing that first vital contact with the newborn through the first breastfeed. I also saw the extent to which breastfeeding was neglected in the medical and paediatric curricula.

*Q: Was the topic not covered?*

A: There were perfunctory references to breastfeeding being ‘important’, but no effort was made to teach us the practical skills we needed to support mothers. Moreover, the general impression was that feeding with formula was an acceptable alternative. Despite the Philippine government having enacted legislation limiting the marketing of milk formula in 1986, I could see that little had changed since my residency days when, as part of the curriculum, we had to memorize the composition of different commercial formulas, and to learn the different water-to-formula ratios needed to mix the different brands. I also remember that we would receive tins of formula on Wednesdays – as strange as that may sound. This was back in 1984, and we just accepted it as normal. When I came back to the Philippines in 1997, I was determined to try to change things. Teaching paediatrics and newborn medicine and attending to high-risk newborns, I saw that we were really not helping, and were in fact getting in the way of the mother and her infant in those first crucial moments.

*Q: In what way?*

A: For example, we were suctioning all newborns to clear their airways as a matter of routine, even though the vast majority didn’t need it. The risk with suctioning is that you expose babies to the risk of low heart rates and hypoxia, potentially traumatize babies’ mouths and throats and compromise their ability to thermoregulate. We were, basically, interfering with natural feeding reflexes that have evolved over the millennia. We were also separating babies from their mothers too early – literally as far as the clamping and cutting of the umbilical cord went – while also taking babies away for weighing or measuring or bathing, exposing the infant to the risk of hypothermia and hypoglycemia. Moreover, we were routinely taking blood through heel pricks for bedside glucose tests, when evidence already indicated that skin-to-skin contact *raises* newborns’ blood sugar levels. We were also exposing infants to the risk of cross-contamination, especially with heel printing for the supposed purposes of identification using ink pads which were hardly ever changed. So, I expressed my concerns about all this and, having trained as a neonatologist abroad, I had some influence among my peers, but I started thinking about what I could do to inspire change across the whole health system.

“We were […] interfering with natural feeding reflexes.”

*Q: What did you focus on?*

A: Back then, there was much discussion of low-cost, evidence-based interventions that could help accelerate progress towards MDGs (Millennium Development Goal) 4 and 5, which, as you may remember, were to reduce child and maternal mortality. Newborn deaths were a particular concern since they comprised roughly half of all under-5 deaths. At that time, the top three causes of newborn death in the Philippines were complications associated with prematurity, asphyxia and sepsis. Focus on the latter intensified in May 2008, when there was an outbreak of neonatal sepsis at a large hospital in Manila that resulted in 25 mostly healthy, full-term babies dying. Among the tragic facts to emerge from the task force investigation into that incident was that not one of the babies who died had been breastfed prior to being whisked off to a nursery.

I had been in dialogue with the WHO country office and, in the aftermath of the sepsis outbreak, I suggested that we initiate a time and motion study in hospitals across the country to find out exactly how newborns were being, so to speak, ‘processed’. The WHO Philippines office supported a Department of Health initiative to observe practices in 51 government hospitals, and we ended up observing just under 500 deliveries. We found that babies were being subjected to precisely the unhelpful actions I just described. It was out of that formative research that we developed the Essential Intrapartum Newborn Care (EINC) protocol that became national health policy in 2009. For newborns, immediately after birth, a four-step timebound sequence was recommended and promoted as *Unang Yakap *(First Embrace) in a social marketing campaign.

*Q: Can you describe Unang Yakap in simple terms?*

A: It comprises four steps, designed to be executed in sequence. The first step is immediate and thorough drying of the baby, as opposed to removal from the mother and bathing, followed by early skin-to-skin contact between the mother and the newborn. This has both psychological and physiological impacts. For reasons we don’t fully understand, skin-to-skin contact raises babies’ blood sugar levels, helping to get them through those early moments. Other benefits include better thermoregulation, exposure to maternal skin flora, and the triggering of suckling and let-down reflexes in the mother and her baby. Step three is properly-timed cord clamping, and step four is non-separation of the mother and baby to allow for early breastfeeding initiation and of course maternal-infant bonding.

*Q: Why is the timing of cord clamping so important?*

A: Because the umbilical cord pulsations continue in those first minutes, delivering not just oxygen-laden red blood cells, but also white cells and pluripotent stem cells which are essential for the newborn’s maturing immune system. In the moments when a baby's lungs are not yet functioning efficiently as gas exchange organs, there is oxygen and sugar coming directly from the mother. So just by delaying the cutting and clamping of the cord for one minute, you make these babies warmer and healthier.

“We really needed to teach […] a new ‘dance’”

*Q: How easy was it to get health workers to change their practices?*

A: It was challenging. We really needed to teach doctors, midwives and nurses a new ‘dance’ and, in some instances, insert steps to ensure that the sequence was followed. For example, by having the nurse give the mother the prescribed intramuscular injection of oxytocin to make the uterus clamp down after putting mother and baby skin to skin, we helped nurses to unlearn too-early cord clamping. It really is a kind of choreography.

*Q: You clearly had significant success with the introduction of the EINC protocol and First Embrace. Why did you then decide, in 2010, to set up a non-profit dedicated to the health of the mother and child?*

A: I came to the view that setting up a separate non-profit entity would facilitate the health-system work that we had needed to undertake. *Kalusugan ng Mag-ina *(KMI) which translates into Health of Mother and Child, was set up as a network of experts based in the Philippines, which could act as facilitators guiding efforts to advance EINC and other breastfeeding agendas through clinical practice guideline development, policy reform and capacity building. We had something of a baptism of fire in the early years, coming through numerous health emergencies including, in November 2013, super typhoon Haiyan, which severely disrupted health service delivery for birthing mothers and their newborns. KMI participated in the post-Haiyan nutrition cluster and rehabilitation response, highlighting the value of EINC in the post-disaster context. We went on to establish ourselves as a trusted NGO working to promote and protect the health of the mother-child dyad in the country and across the region. At home, in collaboration with UNICEF, our work includes contributing to Department of Health capacity building and health financing initiatives for the community-managed Philippine Integrated Management of Acute Malnutrition programme. Across the region, we have collaborated with the FHI 360 Alive and Thrive programme to establish Breastfeeding Centres of Excellence in Cambodia, Lao People’s Democratic Republic, Myanmar and Viet Nam.

*Q: What are your priorities going forward?*

A: A core KMI aim was to raise the baseline breastfeeding initiation rates, in view of the tapering off that typically happens after the first 4-6 months to a year. According to the National Demographic and Health Survey 2022, our breastfeeding initiation rate currently stands at 54.1%, but that drops to an exclusive breastfeeding rate of 40.9% among 0-5-month-olds. Ideally, we’d be a good deal higher.

*Q: WHO guidance is for exclusive breastfeeding to six months. Do you feel this is inadequate?*

A: It’s true that WHO recommends exclusive breastfeeding until six months, but WHO and UNICEF also recommend that breastfeeding be continued up to two years or beyond. So, there is mixed messaging on this. Personally, I feel that the WHO/UNICEF guidance is not given enough emphasis.

*Q: What are the main challenges to further improving breastfeeding rates?*

A: The headwinds we face include lack of capacity in the health system, most notably in terms of health human resources, unsupportive work policies including short parental leave, and persistent countervailing cultural norms. There is this attitude that motherhood should be hard. This feeds through to a lack of support in the home, community and in the workplace. And, of course, the persistent efforts of artificial milk formula manufacturers who continue to aggressively market their products, exploiting loopholes in decades-old milk codes. These are of course enormous challenges, and my message as we lead up to World Breastfeeding Week is that nothing will change if the state does not take a lead and start investing in mothers.

